# Toward an Accurate IR Remote Sensing of Body Temperature Radiometer Based on a Novel IR Sensing System Dubbed Digital TMOS

**DOI:** 10.3390/mi13050703

**Published:** 2022-04-29

**Authors:** Moshe Avraham, Jonathan Nemirovsky, Tanya Blank, Gady Golan, Yael Nemirovsky

**Affiliations:** 1 Department of Electrical Engineering and Electronics, Ariel University, Ariel 40700, Israel; smoa@technion.ac.il (M.A.); gadygolan@gmail.com (G.G.); 2 Electrical and Computer Engineering Faculty, Technion—Israel Institute of Technology, Haifa 32000, Israel; jnemirov@technion.ac.il (J.N.); tblank@technion.ac.il (T.B.)

**Keywords:** body temperature, digital TMOS, COVID-19, infrared thermometer, forehead temperature, skin emissivity, thermography, thermometry

## Abstract

A novel uncooled thermal sensor based on a suspended transistor, fabricated in standard CMOS-SOI process, and released by dry etching, dubbed Digital TMOS, has been developed. Using the transistor as the sensing element has advantages in terms of internal gain, low power, low-cost technology, and high temperature sensitivity. A two channel radiometer, based on the new nano-metric CMOS-SOI-NEMS Technology, enables remote temperature sensing as well as emissivity sensing of the forehead and body temperatures of people, with high accuracy and high resolution. Body temperature is an indicator of human physiological activity and health, especially in pediatrics, surgery, and general emergency departments. This was already recognized in past pandemics such as SARS, EBOLA, and Chicken Flu. Nowadays, with the spread of COVID-19, forehead temperature measurements are used widely to screen people for the illness. Measuring the temperature of the forehead using remote sensing is safe and convenient and there are a large number of available commercial instruments, but studies show that the measurements are not accurate. The surface emissivity of an object has the most significant effect on the measured temperature by IR remote sensing. This work describes the achievements towards high–performance, low-cost, low power, mobile radiometry, to rapidly screen for fever to identify victims of the coronavirus (COVID-19). The main two aspects of the innovation of this study are the use of the new thermal sensor for measurements and the extensive modeling of this sensor.

## 1. Introduction

### 1.1. The Role of IR Remote Temperature Measurements

IR Radiation thermometry is attractive in many challenging temperature measurement situations because it is a non-contact, non-intrusive, and fast technique. Thermal radiation is governed by the fundamental physical laws established over one hundred years ago by Kirchhoff, Stefan, Boltzmann, Wien, and Planck. These laws directly link emitted blackbody radiation, totally or spectrally resolved, to the thermodynamic temperature of the emitting source. Actual practical measurements by radiation thermometry, however, are prone to several uncertainties associated with, for example, surface emissivity and environmental effects such as ambient temperature and reflected ambient radiation [1,2].

### 1.2. The Role of Emissivity

The recent COVID-19 pandemic has increased the need and motivation for accurate and low-cost thermometers that detect human body temperature with a high accuracy of 0.1 °C. However, a deeper understanding of the physics of the topic under study teaches us that this accuracy cannot be achieved without determining the surface emissivity as well. This defines the main research question of the present study: how we measure accurately the surface emissivity, as well as the surface temperature.

Emissivity has a coefficient from 0 to 1 that is the ratio of the emitted energy of an object to that of a theoretical blackbody at the same temperature. All infrared (IR) thermometry is based on the concept of the blackbody, which eventually leads mathematically to the concept of emissivity. Planck’s law provides the only theoretically rigorous link between the temperature of a blackbody and a radiated energy of precise quantifiable characteristics. However, emissivity is an elusive parameter. It makes sense only for gray bodies; a gray body is an imperfect blackbody, i.e., a physical object that partially absorbs incident electromagnetic radiation. The ratio of a gray body’s thermal radiation to a blackbody’s thermal radiation at the same temperature is called the emissivity of the gray body. The emissivity of the gray body does not depend on the wavelength for a given range of optical bandpass [1].

### 1.3. The Human Body Temperature and Emissivity

Based on numerous studies, it is now established that many factors affect the body temperature and emissivity, in the presence of extrinsic (i.e., hot environments) or intrinsic factors (i.e., pathology, exercise), such as: ambient temperature; exercise; sweat; blood pressure; superficial blood flow; heat conduction from deeper tissues (including muscle); and heat loss across the skin’s surface. The skin is also the site of reciprocal heat transfer between the human body and the external environment. The extent to which heat transfer occurs is largely dependent on environmental conditions, such as ambient temperature, water vapor, and the thermal properties of human skin [3,4,5,6,7,8]. The following facts are now clear:

The human body controls core body temperature within a tight band typically between 36 and 38 °C, despite varying ambient temperatures. Thermoreceptors located within the hypothalamus, spinal cord, skin, and some abdominal organs monitor temperature changes, and work via negative feedback to initiate autonomic mechanisms to either conserve (via shivering and vasoconstriction) or lose (via sweating and vasodilation) body heat [9].

In summary, the forehead skin emissivity is mainly determined by the water contents. In the optical bandpass of ~5–14 µm it is around 0.98, regardless of the ethnicity or gender. Living people’s temperature may change only in the range of 32–42 °C; the emissivity may change more or less by ±0.005 around 0.98 (see Appendix B).

## 2. The Building Block of the Radiometer under Study

### 2.1. The Digital TMOS

In recent years, there have been tremendous developments in instrumentation. For instance, infrared focal plane arrays can now produce images with a spatial resolution of the order of 10 mm with a temperature resolution of 0.01 K. However, these imagers cannot detect the human body temperature correctly by remote sensing. As explained above, a radiometer that detects both temperature and emissivity is required. Therefore, a two-channel sensing system is required. The preferred IR sensor for the radiometer should be linear, reproducible, sensitive to small temperature changes, accurate, and manufactured with a mature technology that requires low power during operation (see Section 3).

**Figure 1:**
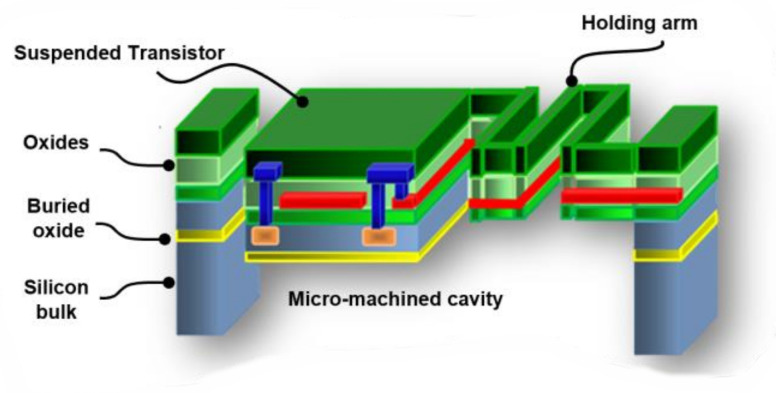
Schematic description of the TMOS.

The TMOS, short for Thermal-MOS, is a thermal sensor based on a thermally insulated MOSFET transistor. The TMOS is manufactured by the CMOS-SOI and MEMS process. A schematic description of the analog TMOS is shown in Figure 1.

The micro-machined thermally insulated transistor has very low thermal mass and very low thermal conductivity. The absorbed photons increase the TMOS temperature and modify the current–voltage characteristics, and since the transistor is operated at a subthreshold region, its I-V characteristics are highly dependent on temperature (exponentially), therefore a highly sensitive sensor is achieved [10,11,12,13,14,15,16,17].

**Figure 2:**
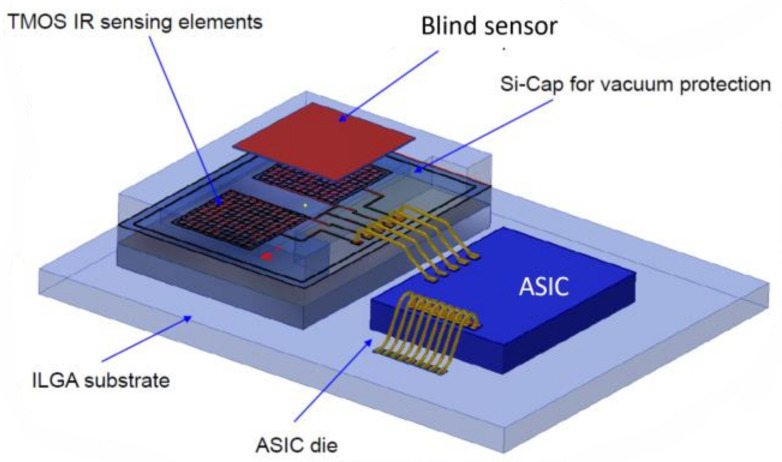
Schematic description of the digital TMOS. ASIC stands for Application Specific Integrated Circuit.

The Digital TMOS is described in Figure 2.

The sensor is based on mosaic design, and it is differential: it contains a blind sensor with identical design, which is covered by mirror. Therefore, the DC current is canceled by differential reading. The electrical signal is read and processed on the ASIC.

**Figure 3:**
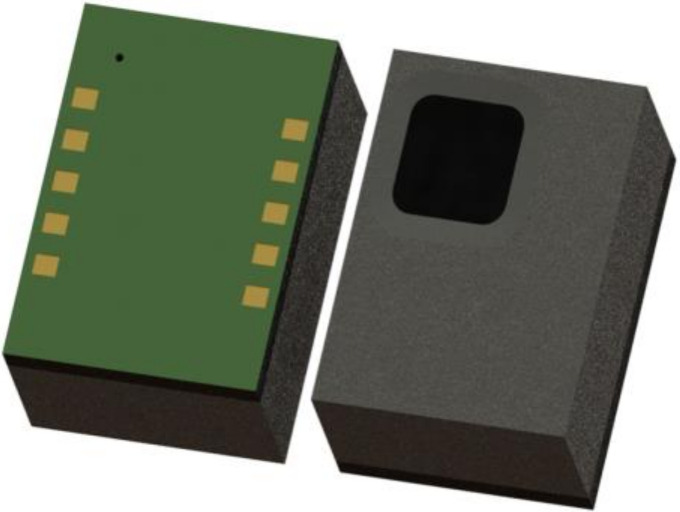
Vacuum Packaged Digital TMOS with Organic Land Grid Area (LGA) with Surface Mount Technology (SMT). The packaged device dimensions: 3.2 × 4.2 × 1.45 mm^3^.

The packaged device is available commercially [18] and is shown in Figure 3.

**Figure 4:**
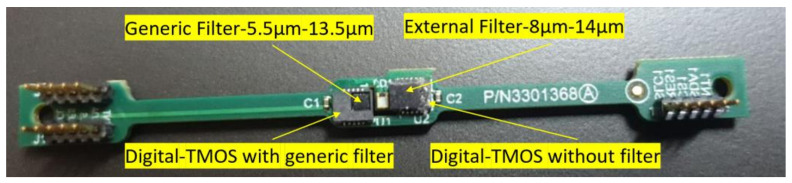
Image of the device under study with two channels.

The two-channel radiometer under study contains two digital TMOS units, each TMOS is covered with a different filter, first, a generic filter of 5.5–13.5 µm (wide bandpass filter), and the second one with an external filter with a bandwidth of 8–14 µm (narrow bandpass filter). The device is shown in Figure 4.

### 2.2. The Radiometer under Study

#### 2.2.1. With No Optics

**Figure 5:**
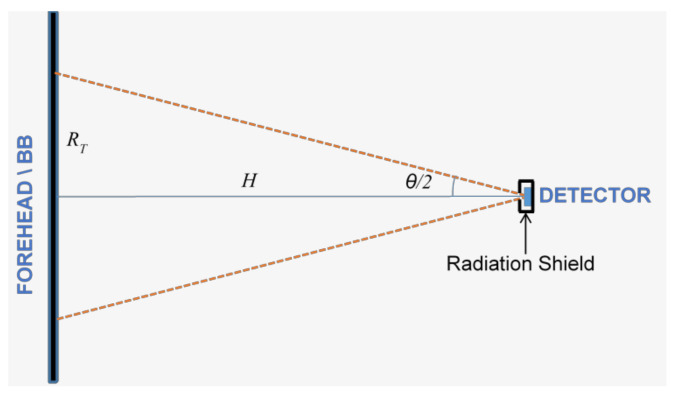
Radiometer with no optics.

The detector (without lens) is placed in front of a forehead (Figure 5). In the case of calibration, the detector views only a large and uniform emitting surface of a blackbody, instead of a forehead, without any chopping.

Parameters:

- H—distance between a target (forehead/blackbody) and a detector;
- A_D_—detector area;
- A_T_—target area;
- t_filter_—optical filter transmittance in a wavelengths range λ1–λ2 or λ3–λ4;
- θ/2—Half Field-of-View (FOV);
- ε–object target emissivity;
- W_λ1–λ2_ (T_BB_)—blackbody emitting power according to Planck’s Law in a given optical bandpass;
- η—absorption coefficient

The power incident absorbed by the detector from the target is equal to the object-emitted power and the reflected power from the ambient environment by the object:

(1) $$\begin{array}{c} P_{\lambda 1-\lambda 2}\left(\varepsilon ,\;T_{obj},T_{amb}\right)=\underset{emitted\;by\;the\;object}{\underbrace{\varepsilon_{obj}A_{D}\cdot W_{\lambda 1-\lambda 2}\left(T_{obj}\right)\cdot t_{filter}\cdot \eta \cdot \sin^{2}\left(\frac{\theta }{2}\right)}}+\\ \underset{reflected}{\underbrace{\left(1-\varepsilon_{obj}\right)\cdot A_{D}\cdot W_{\lambda 1-\lambda 2}\left(T_{amb}\right)\cdot t_{filter}\cdot \eta \cdot \sin^{2}\left(\frac{\theta }{2}\right)}}\\ =PTF\cdot \varepsilon_{obj}W_{\lambda 1-\lambda 2}\left(T_{obj}\right)+PTF\cdot \left(1-\varepsilon_{obj}\right)\cdot W_{\lambda 1-\lambda 2}\left(T_{amb}\right) \end{array}.$$

where *PTF* is the Power-Transfer-Function. The proof for the emitted power which is absorbed by the sensor (the first term) is described in Appendix A.

In the above modeling, we have assumed that the blackbody is larger than the target area (see Figure 5). Under this condition, the *PTF* depends only on *A_D_*, and the Field of View (FOV). The distance is not a parameter. Furthermore, the PTF varies for different units, but its exact value is determined during the calibration process (see Section 4).

From Expressions (1) it is obvious that there are two unknowns: the object temperature, and emissivity. Hence, two devices are required, with different optical bandpass filters, defined by λ1–λ2 and λ3–λ4:

(2) $$P_{\lambda 1-\lambda 2}\left(\varepsilon ,\;T_{obj},T_{amb}\right)=PTF\cdot \varepsilon W_{\lambda 1-\lambda 2}\left(T_{obj}\right)+PTF\cdot \left(1-\varepsilon \right)\cdot W_{\lambda 1-\lambda 2}\left(T_{amb}\right)$$

$$P_{\lambda 3-\lambda 4}\left(\varepsilon ,\;T_{obj},T_{amb}\right)=PTF\cdot \varepsilon W_{\lambda 3-\lambda 4}\left(T_{obj}\right)+PTF\cdot \left(1-\varepsilon \right)\cdot W_{\lambda 3-\lambda 4}\left(T_{amb}\right)$$

In this study we are using the following bandpass optical filters, termed narrow and wide, respectively: narrow bandpass λ1–λ2 = 8–14 µm and wide bandpass λ3–λ4 = 5.5–13.5 µm.

#### 2.2.2. With Optics Limiting the Field of View (FOV)

**Figure 6:**
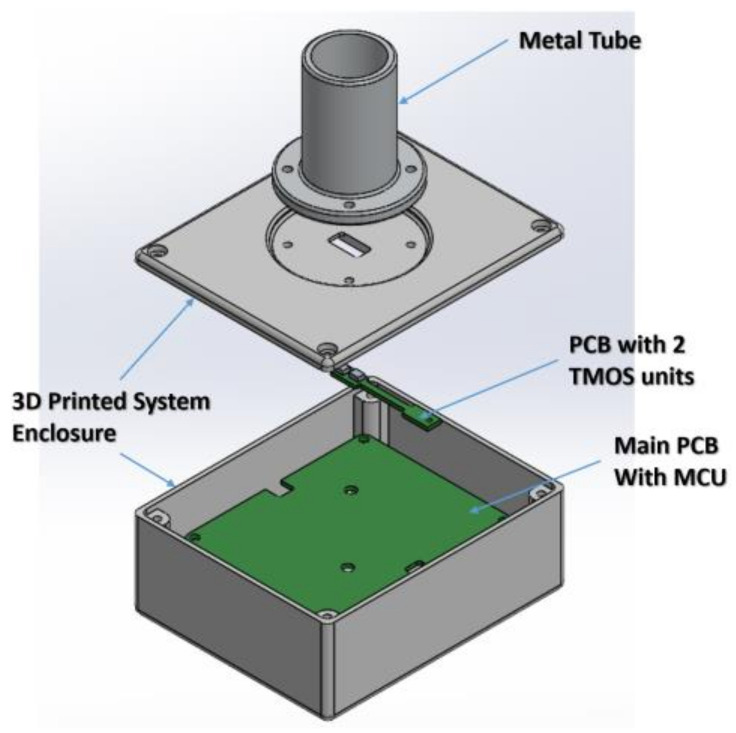
The schematics of the packaged radiometer, with a tube that limits the Field of View.

**Figure 7:**
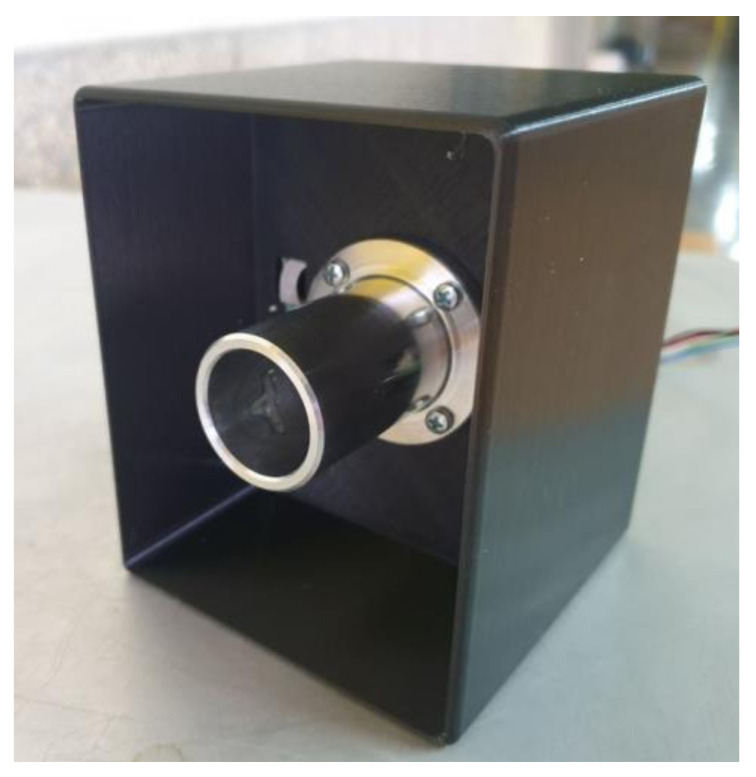
A photo of the radiometer under study.

The reflected (second term) of Expressions (1) may introduce significant errors unless the field of view is limited. Figure 6 presents the schematic approach of this study on how to limit the FOV, while Figure 7 exhibits an image of the radiometer.

The edge of the metal tube is placed very close, 1–2 cm, from the forehead or the blackbody during calibration. An appropriate lens placed inside the tube is optional and reduces the effect of distance between sensor and forehead.

## 3. Modeling and Calibration

### 3.1. Modeling

**Figure 8:**
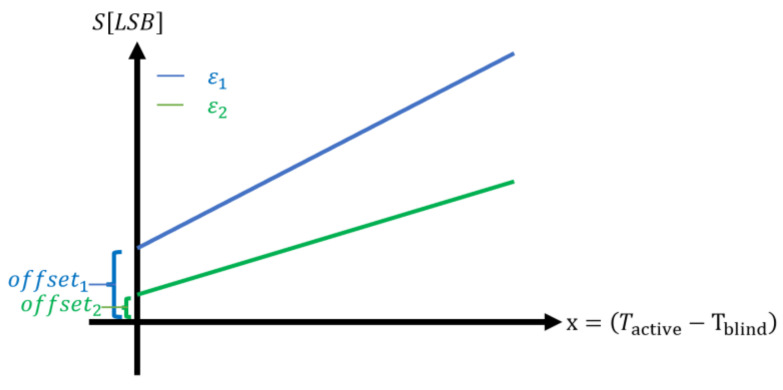
Schematic presentation of the measured signal, illustrating the gain and the offset. The gain is the slope of the line.

The model is based on empirical measurements. We can refer to it as phenomenological or heuristic. We measure the temperature difference between the active pixel to the blind pixel (see Figure 2), multiply it by the gain and then add the offset (see Figure 8):

(3) $$S\left[LSB\right]=gain\cdot \underset{\Delta T_{measrued}}{\underbrace{\left(T_{active}-T_{blind}\right)}}+offset$$

Figure 8 describes schematically the measured signal as a function of the temperature difference and with two different object emissivity, ε_1_ > ε_2_.

The offset is measured when there is no temperature difference between the active and blind pixel, ∆T_measured_ = 0. Ideally, the output signal should be zero. In practice, every sensor, regardless of its specific nature, exhibits offset. The offset depends on the object temperature, emissivity, and ambient temperature. Since the measured plot is very stable in time for a given set of parameters, the offset can be calibrated.

**Figure 9:**
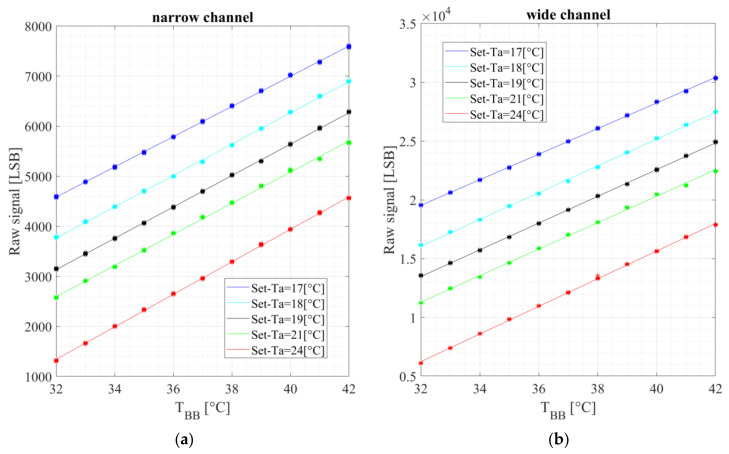
The measured signal in the lab as a function of the object temperature (or blackbody temperature) for different ambient temperatures measured by the air condition (Ta); The dots are the measured raw data, and the lines are the linear fit: (**a**) narrow channel bandpass 8–14 µm; (**b**) wide channel bandpass 5.5–13.5 µm.

Figure 9 exhibits a typical measured signal, at an ambient temperature of 20 °C as a function of object temperature.

The modeling is based on the observed linearity.

### 3.2. Calibration

**Figure 10:**
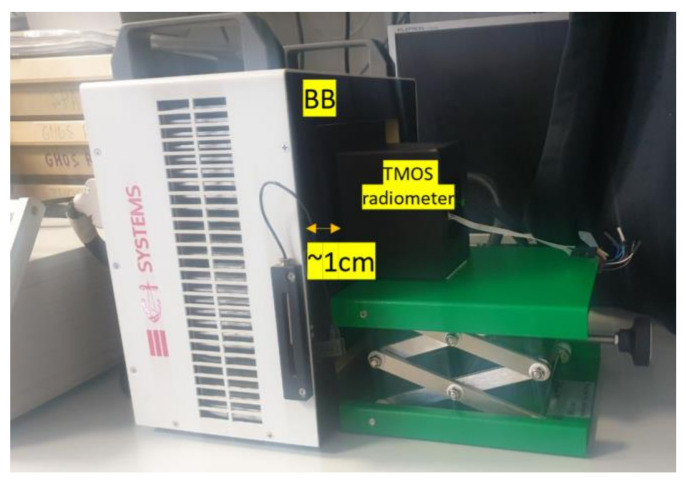
The calibration setup.

The calibration setup is shown in Figure 10.

The calibration is based on a commercial, extended area blackbody [19], instead of a cavity blackbody. Each channel of the radiometer is calibrated between blackbody temperatures of 32 °C and 42 °C, for at least five different ambient temperatures. Ambient temperatures are determined by the Proportional-to-Absolute-Temperature (PTAT) circuit on the ASIC. Accuracy of ΔT = 0.1 °C and better than Δε = 0.005 is achieved.

**Figure 11:**
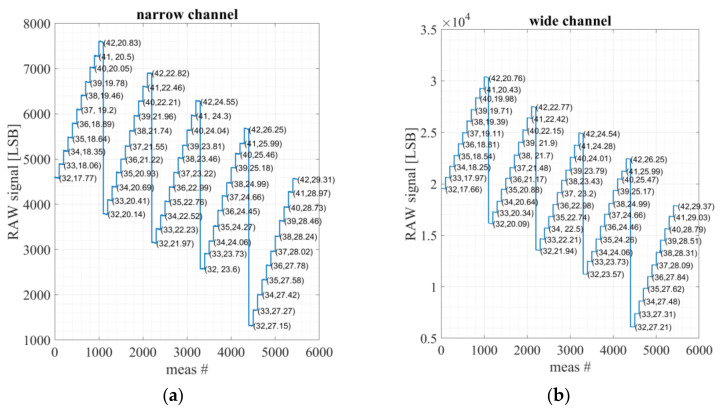
The measured raw data of the signal for blackbody temperature range of 32 °C–42 °C, for five different ambient lab temperatures. The measurement rate is 8 Hz, near each blackbody temperature measurement the average ambient temperature (measured by the TMOS ASIC PTAT) is presented by the format (T_blackbody_,T_ambient_) in Celsius degrees: (**a**) narrow channel bandpass 8–14 µm; (**b**) wide channel bandpass 5.5–13.5 µm.

The ambient temperature has a strong effect on the results as shown in Figure 11.

The measurements are analyzed according to Appendix C, where the reference value is determined by the blackbody. The accuracy and precision for the calibration are shown in Table 1.

## 4. Measurements Results

### 4.1. Calibration Validation

The results and the modeling are validated with the same blackbody, where we now measure at arbitrary testing points not included in the calibration. The accuracy and precision for the validation of calibration are shown in Table 2.

### 4.2. Forehead Measurements with Different of Cosmetics and Ointments

The emissivity of human skin has acquired considerable importance because of the increasing use of fever screening for elevated temperatures associated with COVID-19 and other illnesses or to consider the effect of applied cosmetics or ointments [20,21].

We applied common creams, cosmetics, lotions, and ointments on the forehead, and then compared the measured surface temperature with the commercial non-imaging no-contact IR thermometers described in Section 5. The applied materials included common make up, moisturizing creams, and skin treatments with vitamin C serum, collagen-based serum and retinol-based serum. As expected, the Digital TMOS radiometer was more sensitive to the changes in surface temperature due to the change in emissivity. The Digital TMOS radiometer exhibited a reduced surface temperature, up to 2 °C, for healthy people and certain cosmetics. Cosmetics and topical medication, which increase the water content in the forehead, increase the emissivity. Since we measured the emitted power by the surface, the increase in emissivity resulted in a decreased surface temperature. Small changes in the emissivity, of the order of several tenth of percentage, already introduced changes in the surface temperature. Unless emissivity is taken into consideration, the IR non-contact temperature measurement can be unreliable and mask people with fevers. This aspect of our study will be reported elsewhere, once a large measurement of the data analyzed with statistical techniques is obtained.

### 4.3. Correlation between Forehead Measurements and Core Body Temperature—A Disclaimer

The public is familiar with body thermometers (such as mercury thermometers). Hence, all commercial IR remote temperature sensors introduce an offset (a correlation between body and surface temperature). The radiometer reported here is remarkable since it models the offset by using the emissivity in addition to the object’s and the ambient temperatures. We do not present here the offset correction tailored to the Digital TMOS since a large measurement of the data analyzed with statistical techniques is still required.

## 5. Comparison to Other State-of-the-Art Commercially Available IR Remote Temperature Thermometers

Table 3 summarizes the proposed microsystem performance and compares it with commercial state-of-the-art non-imaging, no-contact IR thermometers. The values are taken from the datasheets of the commercial products. Because of the digital nature of the measurements and the lack of uniform terminology, we follow the terms defined in the Appendix C.

**Figure 12:**
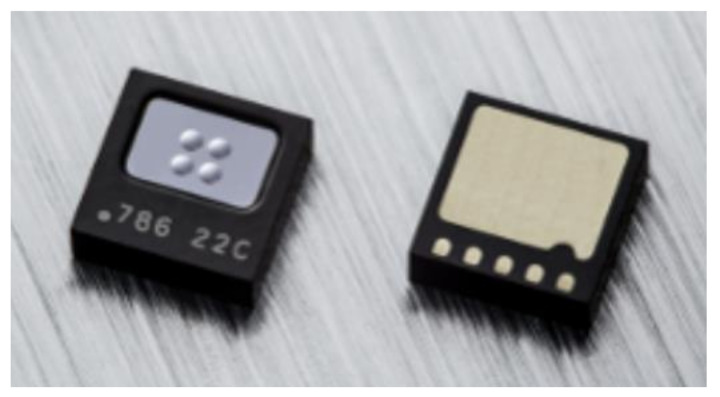
MLX90632 [22].

**Figure 13:**
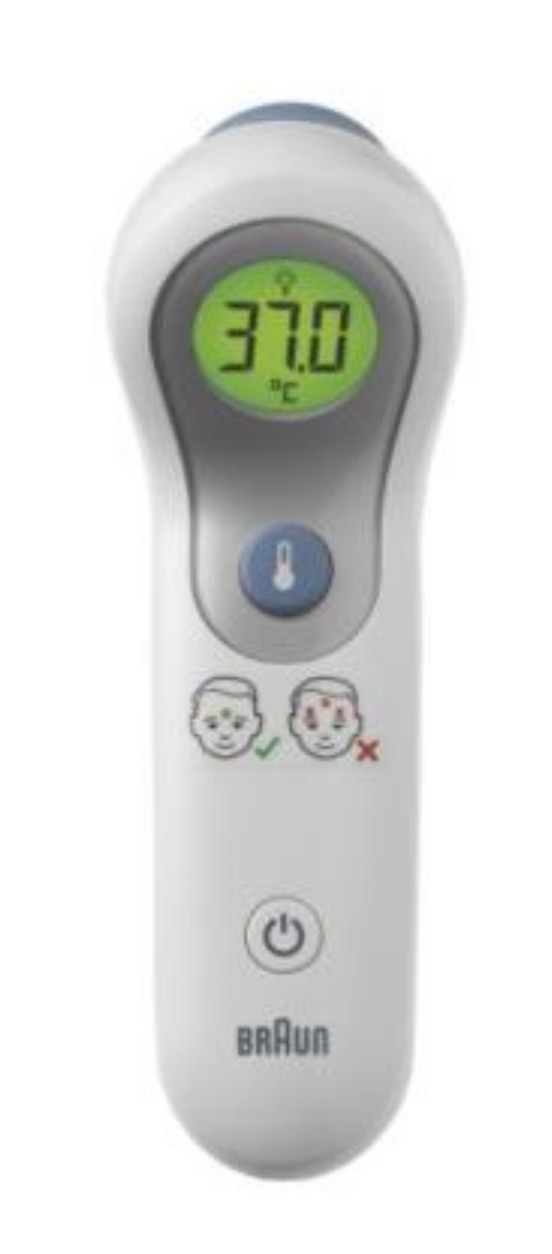
BRAUN NTF3000 [23].

**Figure 14:**
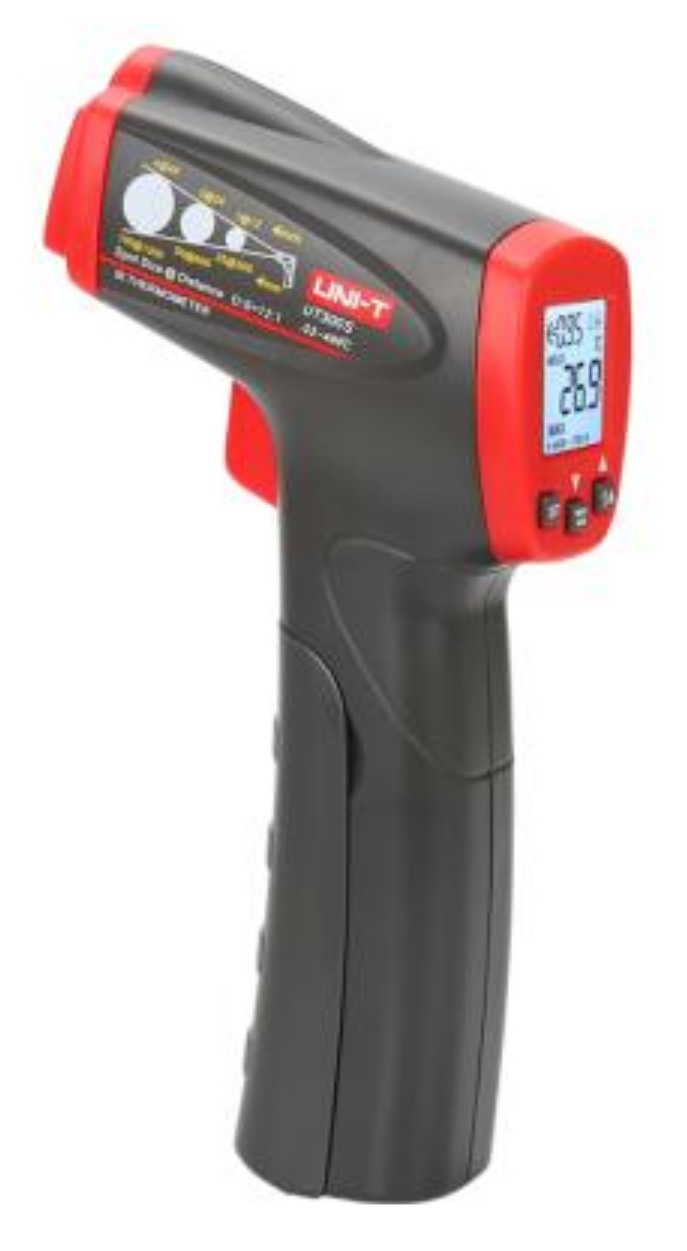
UT300S Infrared Thermometer [24].

The product is shown in Figure 12:

The product is shown in Figure 13:

The product is shown in Figure 14:

## 6. Summary and Conclusions

Forehead temperature measurement using an infrared thermometer may be used to safely and rapidly screen for fever to identify victims of COVID-19. Unless both emissivity and temperature are measured, the screening may fail [3,25]. The temperature of the forehead is known to be highly correlated with the internal body temperature and it is easier to access the forehead temperature than the tympanic (ear) temperature [8].

A two-channel radiometer, based on the new nano-metric CMOS-SOI-NEMS Technology, dubbed Digital TMOS [18], enables remote temperature sensing as well as the emissivity sensing of forehead and body temperature of people, with high sensitivity, accuracy, and high resolution.

Currently it is intended for indoor application and for living human bodies with temperatures in the range of 32–42 °C and forehead emissivity around 0.98. We have emphasized the role of emissivity in determining the forehead surface temperature. We have shown that an accuracy as well as a precision better than 0.005 in the emissivity is required to achieve an accuracy of 0.1 °C in the forehead surface temperature.

The Digital TMOS radiometer outperforms commercial gun thermometers or the medical grade sensors, which are based on one IR non-contact sensor. The commercial instruments assume for the user a nominal emissivity, whereas the Digital TMOS radiometer measures both emissivity and temperature with the required fidelity.

We have shown that common cosmetics as well as the measuring lab ambient temperature and body exercise may affect the forehead surface emissivity. Since the accuracy with which we can determine the temperature is strongly dependent on the accuracy with which we can measure the emissivity of the surface, a two-channel radiometer is essential.

Non-contact temperature measurement, known also as “dual channels” or “two-color” IR thermography, has been applied for many years. The different non-imaging commercial instruments use different thermal sensors such as pyroelectric, thermoelectric (thermopiles), and resistive sensors [26]. The most recent development in this family of thermal sensors is the Digital TMOS based on CMOS micro- or nano-machined transistor, vacuum packaged, and combined with CMOS ASIC, where ambient temperature measurement and digital signal processing are performed. This new technology with advanced processor technology increases the system’s stability, reliability, resolution, and speed.

The authors have measured and compared the performance of the Digital TMOS radiometer with state-of-the-art commercial non-imaging IR non-contact thermometers. The measured results exhibit the potential of the Digital TMOS to outperform the existing products.

Forehead temperature measurement using an infrared thermometer may be used to rapidly and safely screen for fever to identify victims of COVID-19. However, a large measurement of the data analyzed with statistical techniques is still required. A clinical study in hospitals is planned to meet this requirement.

Furthermore, this study compares commercial IR thermometers in stable, resting, laboratory conditions. We intend to extend the Digital TMOS radiometer to outdoor measurements.

The role of calibration in any MEMS sensing system is well-known. Calibration determines not only the accuracy but also the cost of commercial products. The role of emissivity in remote temperature measurements is also well established. Detailed information critical to these topics are typically kept private by manufacturers. The academic work reported here focuses on the above topics, hoping to inspire additional studies in the open literature.

## Figures and Tables

**Table 1:** Calibration of accuracy and precision.

	Temperature		Emissivity	
Number of Measurements Averaged	Accuracy (Mean)	Precision (Standard Deviation)	Accuracy (Mean)	Precision (Standard Deviation)
1	0.1267	0.103	0.0013	0.001
8	0.0806	0.0531	7.43 × 10^−4^	5.11 × 10^−4^
32	0.0739	0.0413	6.50 × 10^−4^	3.87 × 10^−4^
100	0.0704	0.0376	5.99 × 10^−4^	3.56 × 10^−4^

The values depend on the average number of measurements.

**Table 2:** Validation of calibration accuracy and precision.

	Temperature		Emissivity	
Number of Measurements Averaged	Accuracy (Mean)	Precision (Standard Deviation)	Accuracy (Mean)	Precision (Standard Deviation)
1	0.1218	0.0935	0.0012	9.49 × 10^−4^
8	0.0725	0.051	6.75 × 10^−4^	5.01 × 10^−4^
32	0.0666	0.0351	5.87 × 10^−4^	3.42 × 10^−4^
100	0.0664	0.028	5.79 × 10^−4^	2.68 × 10^−4^

**Table 3:** Performance comparison of the radiometer understudy and commercial products that are considered state-of-the-art. The superscripts in the table are elaborated in the tables below for each product (referred to as the Notes table). The “channel” refers to the optical bandpass.

Parameter	This Work: Wide Channel [18]	Commercial Medical Grade [22]	Commercial, No Touch Forehead Thermometer [23]	Commercial Gun Thermometer [24]
Notes Table	See Table 4	See Table 5	See Table 6	See Table 7
Concept	2 channels ^a^	1 channel ^a^	1 channel ^a^	1 channel
Packaged Device (mm^3^)	3.2 × 4.2 × 1.45 ^b^	3 × 3 × 1	Gun thermometer	Gun thermometer
Factory Calibrated	Yes	Yes	Yes	Yes
Field of View (°)	80 ^c^	50	N/A	5
Temp. Sensitivity	2000 (LSB/K)	NETD ^b^ = 50 mK	N/A	N/A
Noise (peak to peak)	120 (LSB)]	N/A	N/A	N/A
Resolution (°C)	0.02	0.01	0.1	0.1
Optical Filter (µm)	5.5–13.5	2–14	N/A	8–14
Accuracy (°C)	0.07 ^d^	0.2 ^c^	0.2	±2
Precision (°C)	0.04 ^d^	N/A	N/A	±0.5
Supply Voltage (V)	1.7–3.6	3.3	2 AA battery	9
Supply Current	10 uA	1 mA ^d^	N/A	N/A
Total Power Consumption	0.036 mWatt	3.3 mWatt	N/A	N/A
Ambient temp. range (°C)	15–30 ^e^	15–40 ^c^	15–40 ^b^	N/A
Object temp. range (°C)	35–42 ^e^	35–42 ^c^	35–42 ^b^	−32~
Storage temperature (°C)	−40–85	−20–85	−25–60	N/A
Response Time	85–125 ms	16 ms–2 s	<2 s	500 ms
Total Power Consumption	0.036 mWatt	3.3 mWatt	N/A	N/A

a–e: Notes for the radiometer understudy (see Figure 3 and Figure 7).

**Table 4:** Notes for the radiometer under study.

Parameter	Note
(a) Concept	The radiometer is based on two channels: wide and narrow optical bandpass filters. Accordingly, it is the only system in Table 4 that yields both emissivity as well as temperature. The wide channel datasheet is specified in STM catalog [18].
(b) Packaged device (mm^3^)	Based on datasheet [18]
(c) Field of View (°)	Based on datasheet [18]. This is the FOV of the sensor without the optical tube. The optical tube further limits the FOV.
(d) accuracy (°C)	Calculated according to Appendix C
(e) Ambient and Object temperature range (°C)	Measured in this study

Notes for Commercial, Medical grade [22]:

**Table 5:** Notes for MLX90632 [22].

Parameter	Note
(a) Concept	The thermal sensor is a CMOS thermopile
(b) Temperature Sensitivity	NETD–Noise Equivalent Temperature Difference–given for refresh rate of 8 Hz and T_obj_ = T_amb_ = 25 °C. Under this condition emissivity does not play any role (see Section 3.2).
(c) accuracy (°C)	From the datasheet [22]: It is very important for the application designer to understand that these accuracies are guaranteed and achievable when the sensor is in thermal equilibrium and under isothermal conditions (no temperature differences across the sensor package).
(d) Supply Current	Supply current of 1 mA at operation and sleep current less than 2.5 µA

Notes for Commercial, No Touch Forehead thermometer [23]:

**Table 6:** Notes for BRAUN NTF3000 [23].

Parameter	Note
(a) Concept	Apparently, there is also a proximity sensor in addition to the IR sensor. Emissivity value is uncontrollable by the user, and is not a parameter.
(b) Ambient and Object temperature range	This temperature gun senses only the body skin or tympanic temperatures. In the datasheet there is a disclaimer: Patient MUST be inside for 30 min before use.

Notes for Commercial, Gun thermometer [24]:

**Table 7:** Notes for UT300S Infrared Thermometer [24].

Parameter	Note
(a) Concept	This gun thermometer is based on a single sensor but addresses both emissivity as well as temperature. The emissivity is assumed and adjusted by the user.

**Figure A1:**
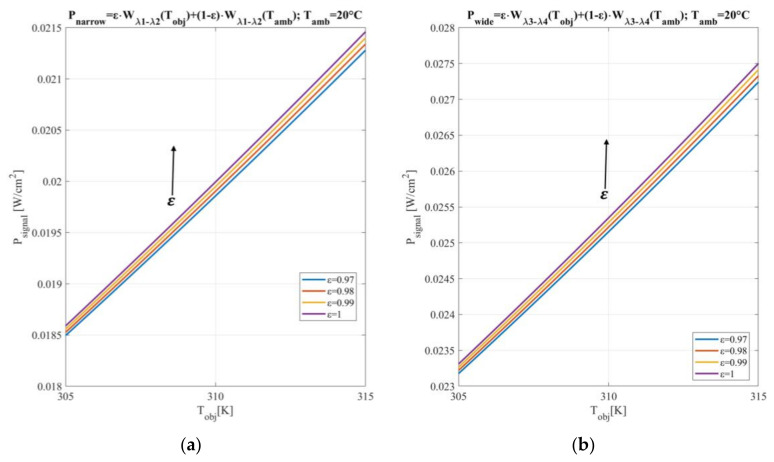
Calculated incident power, emitted and reflected from the object at ambient temperature of 20 °C, for two different bandpass filters; (**a**) narrow bandpass 8–14 µm; (**b**) wide bandpass 5.5–13.5 µm.

**Figure A2:**
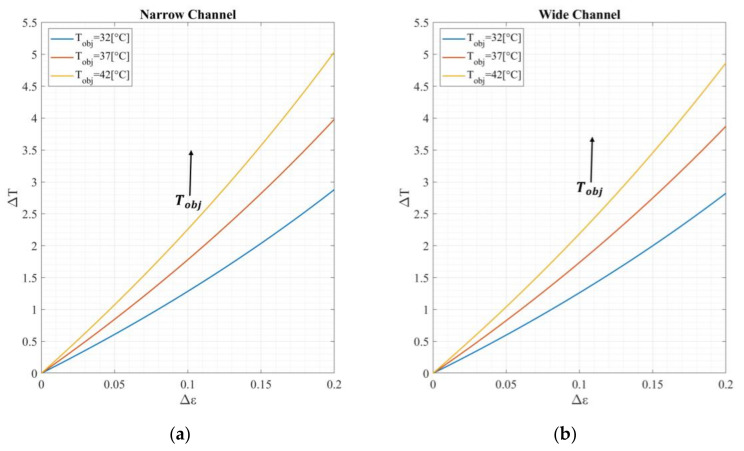
The temperature inaccuracy induced by emissivity inaccuracy up to 0.2 °C at an assumed ambient temperature of 20 °C for the two channels under study: (**a**) narrow bandpass 8–14 µm; (**b**) wide bandpass 5.5–13.5 µm.

**Figure A3:**
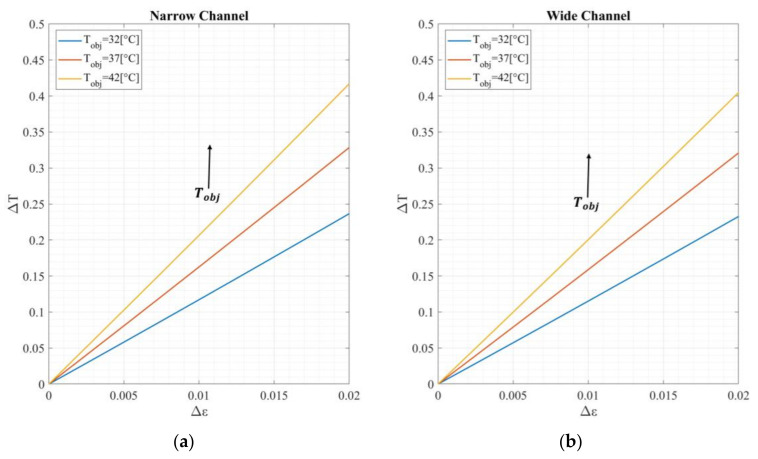
The temperature inaccuracy induced by emissivity inaccuracy up to 0.02 at assumed ambient temperature of 20 °C for the two channels under study: (**a**) narrow bandpass 8–14 µm; (**b**) wide bandpass 5.5–13.5 µm.

**Figure A4:**
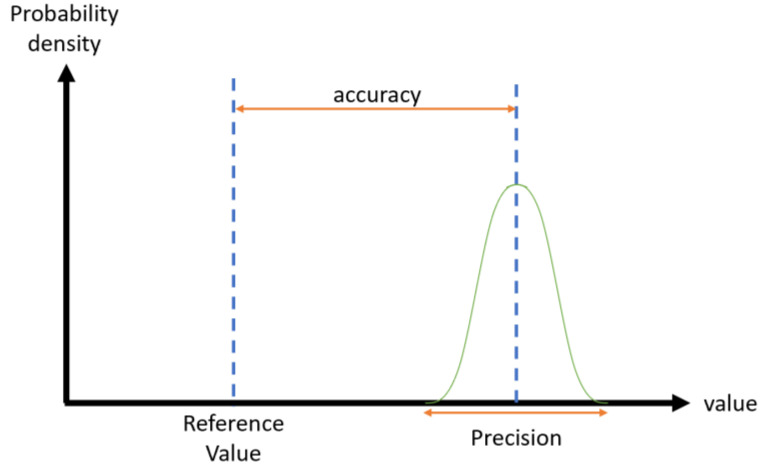
Sensor accuracy and precision.

## Appendix A. Proof for the Absorbed Emitted Power and PTF Definition

The emitted power which is absorbed by the sensor can be expressed by:

(A1) $$P_{\lambda 1-\lambda 2}\left(T_{BB}\right)=\varepsilon A_{T}\cdot W_{\lambda 1-\lambda 2}\left(T_{BB}\right)\cdot \frac{\Omega }{\pi }\cdot t_{filter}\cdot \eta$$

where Ω is the solid angle and the other parameters are described in Section 2.2.1. the target area, *A_T_*, is equal to:

(A2) $$A_{T}=\pi R_{T}^{2}$$

The solid angle is equal to:

(A3) $$\Omega =\frac{A_{D}}{H^{2}+{\left(R_{T}\right)}^{2}}$$

The Half-Field-of-View can be expressed by:

(A4) $$\sin^{2}\left(\frac{\theta }{2}\right)=\frac{{\left(R_{T}\right)}^{2}}{H^{2}+{\left(R_{T}\right)}^{2}}$$

Substituting expressions (A2)–(A4) to (A1) yields:

(A5) $$P_{\lambda 1-\lambda 2}\left(T_{BB}\right)=\varepsilon \cdot \pi R_{T}^{2}\cdot W_{\lambda 1-\lambda 2}\left(T_{BB}\right)\cdot \frac{\frac{A_{D}}{H^{2}+R_{T}^{2}}}{\pi }\cdot t_{filter}\cdot \eta \;=\varepsilon \cdot W_{\lambda 1-\lambda 2}\left(T_{BB}\right)\cdot A_{D}\cdot \frac{R_{T}^{2}}{H^{2}+R_{T}^{2}}\cdot t_{filter}\cdot \eta \;=\underset{\equiv PTF}{\underbrace{A_{D}\cdot \frac{R_{T}^{2}}{H^{2}+R_{T}^{2}}\cdot t_{filter}\cdot \eta }}\cdot \varepsilon \cdot W_{\lambda 1-\lambda 2}\left(T_{BB}\right)=PTF\cdot \varepsilon \cdot W_{\lambda 1-\lambda 2}\left(T_{BB}\right)$$

## Appendix B. Design Considerations

1. *The two approaches for radiometers: the ratio and the power approach*

From Expressions (1) it is obvious that there are two unknowns: the object temperature and the emissivity. Hence, two devices are required, with different optical bandpass filters, defined by λ1–λ2 and λ3–λ4:

(A6) $$P_{\lambda 1-\lambda 2}\left(\varepsilon ,\;T_{obj},T_{amb}\right)=PTF\cdot \varepsilon W_{\lambda 1-\lambda 2}\left(T_{obj}\right)+PTF\cdot \left(1-\varepsilon \right)\cdot W_{\lambda 1-\lambda 2}\left(T_{amb}\right)$$

$$P_{\lambda 3-\lambda 4}\left(\varepsilon ,\;T_{obj},T_{amb}\right)=PTF\cdot \varepsilon W_{\lambda 3-\lambda 4}\left(T_{obj}\right)+PTF\cdot \left(1-\varepsilon \right)\cdot W_{\lambda 3-\lambda 4}\left(T_{amb}\right)$$

Figure A1 presents the calculated incident power of the devices under study, in the temperature range of interest and for various assumed values of emissivity.

Previous reports in the literature recommended the use of the ratio approach, in order to get rid of the emissivity. This is correct only if we neglect the second term. Moreover, the ratio approach shows a normalized temperature sensitivity of only 0.25%, whereas by solving two power equations, the normalized temperature sensitivity is ~1.5%. The normalized temperature sensitivity is obtained from the above simulations, based on A7:

(A7) $$\frac{1}{P_{\lambda 1-\lambda 2}\left(T\right)}\frac{dP_{\lambda 1-\lambda 2}\left(T\right)}{dT}$$

$$\frac{1}{P_{\lambda 3-\lambda 4}\left(T\right)}\frac{dP_{\lambda 3-\lambda 4}\left(T\right)}{dT}$$

2. *The effect of ambient temperature on accuracy*

From expressions (1) and (3) the effect of ambient temperature upon measurement accuracy may be estimated. When the ambient and object temperatures are equal, emissivity does not affect the received signal. If T_obj_ = T_amb_, then the terms with emissivity are canceled. What is reflected cancels what is emitted. It is obvious that, otherwise, measuring the ambient temperature and modeling its effect on the measurements are essential.

For example, assuming T_amb_ = 20 °C, Figure A2 and Figure A3 present the temperature inaccuracy induced by emissivity inaccuracy for different object temperatures.

In practice, for a more realistic emissivity inaccuracy of the forehead of 0.02, it is evident that the inaccuracy in temperature decreases with the decreased object temperature. At T_obj_ = 42 °C, the inaccuracy is~0.42 °C for the narrow channel and ~0.4 °C, as shown in Figure A3.

From Figure A3 the required accuracy in emissivity for a medical grade radiometer can be obtained (see below).

3. *The relation between skin emissivity and temperature*

For the sake of simplicity, we discuss this issue at first for monochromatic radiation.

Monochromatic Planck’s radiation:

(A8) $$W_{\lambda }\left(T\right)=\varepsilon \frac{2\pi hc^{2}}{\lambda^{5}}\cdot \frac{1}{e^{\frac{h\nu }{\lambda kT}}-1}\left[\frac{W}{cm^{2}\mu m}\right]$$

The Temperature differentiation is given by:

(A9) $$\frac{dW_{\lambda }\left(T\right)}{dT}=\varepsilon \frac{2\pi hc^{2}}{\lambda^{5}}e^{-\frac{h\nu }{\lambda kT}}\cdot \frac{hc}{\lambda k}\cdot \frac{1}{T^{2}}$$

Emissivity differentiation:

(A10) $$\frac{dW_{\lambda }\left(T\right)}{d\varepsilon }=\frac{2\pi hc^{2}}{\lambda^{5}}e^{-\frac{h\nu }{\lambda kT}}$$

The relation between emissivity differentiation to temperature differentiation for monochromatic radiation:

(A11) $$\frac{dT}{d\varepsilon }=\frac{\frac{dW_{\lambda }\left(T\right)}{d\varepsilon }}{\frac{dW_{\lambda }\left(T\right)}{dT}}=\frac{\lambda kT^{2}}{\varepsilon hc}=\frac{T}{\varepsilon }\cdot \frac{kT}{\frac{hc}{\lambda }}$$

From Expressions (A11) it can be seen that for monochromatic radiation λ = 10 µm, the object emissivity of 0.98 and object temperature of 310 K and inaccuracy of Δε = 0.01, introduces temperature inaccuracy of 0.6862 °C.

For the two channels Digital TMOS under study, we need to address Figure A3. We observe that the value of the slope ∆T/∆ε at object temperature 37 °C and emissivity of 0.98, is 16.42 and 16.02 for the narrow and the wide channel, respectively. Similarly, the slope ∆T/∆ε at object temperature 42 °C and emissivity of 0.98, is 20.85 and 20.25 for the narrow and the wide channel, respectively. In order to achieve temperature accuracy of 0.1 °C the emissivity should be determined with an accuracy of 0.0048 (see Table A1).

**Table A1:** Relation between temperature inaccuracy to emissivity inaccuracy of narrow and wide filters optical bandpass.

Tobj (°C)	∆T/∆ε Narrow Channel	∆T/∆ε Wide Channel	Required ∆ε for ∆T = 0.1
32	11.835	11.64	0.0084
37	16.42	16.02	0.0061
42	20.85	20.25	0.0048

## Appendix C. The Main Sensor Parameters

The parameters definitions are based on [27].

Temperature sensitivity: (LSB/K) or (DN/K): is the transfer function between the output digital parameter expressed by digital number (LSB or DN) and the input non-electrical parameter (temperature).

Resolution: is the ratio between the output noise level and the sensor sensitivity. It is the minimum detectable non-electrical parameter value under the condition of unitary Signal-to-Noise Ratio (SNR). It is the smallest variation of temperature which provides a detectable output variation. A system with a very low-resolution value is typically referred to as a “High-Resolution System”;

Linearity: proportionality between input and output signals. This parameter is related to the sensor response curve, which correlates the output signal of the sensor to the measurand parameter;

Repeatability: the capability to reproduce output readings for the same value of measurand, when applied consecutively and under the same conditions;

Accuracy: it is the degree of closeness of a measured or calculated quantity to its reference (expected) value, as shown in Figure A4;

Precision: capability to replicate output signals with similar values, for different and repeated measurements, when the same input signal is applied. The precision can be defined as the standard deviation of the calculated quantity from the sensor measurements which were conducted in the same conditions. Figure A4 illustrates precision.

**Noise:** output unwanted signal.
